# Selectively detecting attomolar concentrations of proteins using gold lined nanopores in a nanopore blockade sensor†

**DOI:** 10.1039/d0sc04552g

**Published:** 2020-10-26

**Authors:** Yanfang Wu, Yin Yao, Soshan Cheong, Richard D. Tilley, J. Justin Gooding

**Affiliations:** School of Chemistry, Australian Centre for NanoMedicine, Australian Research Council Centre of Excellence in Convergent Bio-Nano Science and Technology, University of New South Wales Sydney New South Wales 2052 Australia yanfang.wu@unsw.edu.au justin.gooding@unsw.edu.au; Electron Microscope Unit, Mark Wainwright Analytical Centre, University of New South Wales Sydney New South Wales 2052 Australia

## Abstract

Disease diagnosis at earlier stages requires the development of ultrasensitive biosensors for detecting low-abundance biomarkers in complex biological fluids within a reasonable time frame. Here, we demonstrate the development of an ultrasensitive nanopore blockade biosensor that can rapidly diagnose a model protein biomarker, prostate-specific antigen (PSA) with high selectivity. The solid-state nanopores have gold located only along the length of the nanopore whilst the rest of the membrane is silicon nitride. The orthogonal use of materials allows nanopore arrays with a different surface chemistry inside the nanopore relative to the rest of the membrane to be fabricated. The importance of this differential surface chemistry is it can improve the detection limit of nanopore blockade sensors in quantitative analysis. Based on such functionalized nanopore devices, nanopore blockade sensors lower the limit of detection by an order of magnitude and enable ultrasensitive detection of PSA as low as 80 aM. The findings from this study open new opportunities for nanopore sensors in further developments including optical detection and ultralow detection limit biosensing at complex biological fluids.

## Abstract 2

Selective detection of attomolar proteins was achieved using gold lined nanopores in a nanopore blockade sensor.

## Graphical abstract

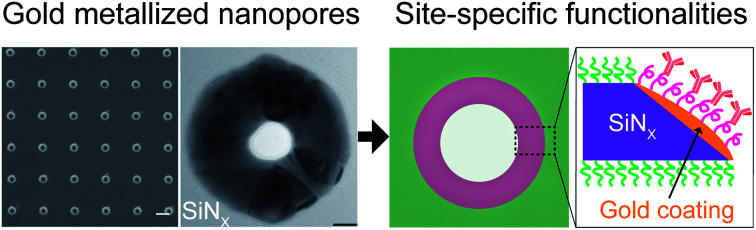

## Introduction

To date, the main method for nanopore sensors has been developed to detect ionic current fluctuations as analyte molecules translocate through the nanopore. With solid state nanopore sensors^1–5^ for single-molecule characterization or quantitative analytical analysis, surface functionalization on nanopore chips is commonly required to enhance specificity for the target species over other species in the sample.^6–8^ In many applications it would be desirable to have a different surface chemistry with the nanopore surface compared with the surrounding membrane, *i.e.*, a surface covering containing specific recognition components in the nanopore and a passivating coating on the membrane to suppress nonspecific adsorption. This is certainly the case for the nanopore blockade sensor we developed to overcome the challenges faced by translocation based nanopore sensors of slow response times and susceptibility to nonspecific signals when employed for quantitative analysis of analytes in low concentrations in complex biological fluids.^9^ The nanopore blockade sensor uses magnetic nanoparticles to decrease response times and reduce detection limits, and blocking of the nanopore to enhance sensor specificity.^9^ The nanopore blockade sensors have been shown to obtain rapid, sub-fM detection of prostate specific antigen (PSA) with high specificity directly from blood.^9^ Surface modification is the key to the enhanced specificity. In the nanopore blockade sensor, antibody modified magnetic nanoparticles capture the analyte of interest and a magnetic field is used to rapidly bring it to a solid-state nanopore array where the magnetic nanoparticle blocks the nanopore to give a signal.^9^ If the magnetic nanoparticle has captured the analyte, PSA in this case, then an immunosandwich is formed with antibodies within the nanopore to give an irreversible blockade event. However, as antibodies are immobilized over the entire membrane surface, PSA can also be captured on the *cis* membrane. It is hypothesized that the performance of this technology could be further improved, as with many nanopore sensor technologies, by only having the recognition species (antibodies) within the nanopores.

To achieve modifying a nanoscale pore with a different surface chemistry to the rest of the membrane is exceedingly challenging. Solid state nanopores are typically modified with surface chemistries composed of polymeric molecules or organosilanes with silica based nanopores^10–12^ or alkanethiols with gold plated nanopores^13^ followed by subsequent conjugation reactions.^8^ However, in most cases the material of the membrane and the solid-state nanopore are the same and hence the surface chemistry modifies both the nanopore surfaces and surrounding membranes. The problem with this of course is that if the surface chemistry in the nanopore is designed to interact strongly with the target species, so will the outer membrane. Alternatively, if the outer membrane is designed to resist interacting with the target species then so will the nanopore. For quantitative analysis neither outcome is positive for the sensor performance. One way to achieve different surface chemistries within the nanopore to the rest of the membrane is to fabricate nanopore chips where the nanopores are composed of a different material to the membrane such that orthogonal surface chemistries can be employed. Such a strategy is in some ways a biomimetic equivalent to a biological nanopore incorporated within a lipid bilayer.^14,15^

Herein, we report a facile and straightforward approach to fabricate solid-state nanopores with metal located only along the length of the nanopore and exploit the fact that the nanopore array and the membrane are composed of different materials to demonstrate a significant improvement in nanopore blockade sensors for ultrasensitive quantitative analysis. We firstly characterize the fabricated gold metallized nanopores with scanning electron microscopy (SEM), transmission electron microscopy (TEM), and dark-field optical microscopy and show the tunability control over the nanopore number, size, and spatial location. We then use an orthogonal self-assembly strategy to selectively modify the metallic nanopore and the surrounding membrane with distinguished functionalities and demonstrate the applicability of functionalized nanopore chips for ultrasensitive detection of PSA, important both for diagnosis and recurrence of prostate cancer. To indicate the importance of differential surface chemistry for nanopore blockade sensors in quantitative analysis, we compare the sensing performance from two kinds of nanopore chips, that is, one modified with site-specific functionalities and the other with a single surface functionality over the whole chip surface. We also investigate the current noise level, the detection limit, and ultrasensitive sensing of 80 aM PSA from whole blood.

## Results and discussion

**Scheme 1:**
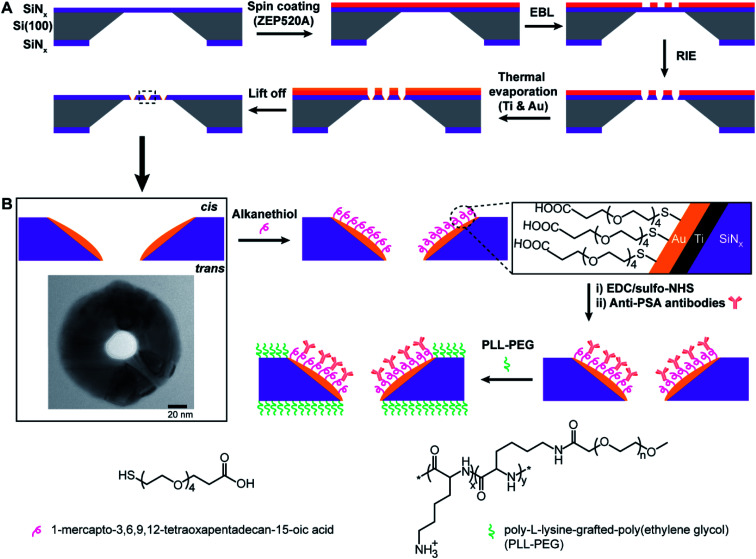
(A) Illustration of fabrication process involving the use of electron beam lithography (EBL), reactive ion etching (RIE) and thermal metal evaporation on electron beam resist ZEP520A coated SiN_*x*_ membrane. Titanium adhesion layer (∼5 nm) and gold film (20–30 nm) are deposited onto nanopore surfaces in high vacuum. Finally, the resist film is lifted off in *N*-methyl pyrrolidone at 80 °C and nanopore chips are cleaned by oxygen plasma treatment. (B) Illustration of surface functionalization process for nanopore chips with spatially defined functionalities. Alkanethiol molecules (*i.e.*, 1-mercapto-3,6,9,12-tetraoxapentadecan-15-oic acid) are employed to specifically modify the entire metallic nanopore followed by the attachment of anti-PSA antibodies *via* EDC/sulfo-NHS reaction. The surrounding SiN_*x*_ membranes outside of metallic nanopores are then passivated by an antifouling polymeric molecule, PLL-PEG. The inset image shows the top view of a gold metallized nanopore on the SiN_*x*_ membrane by transmission electron microscopy (TEM); scale bar, 20 nm. Abbreviations: Ti, titanium; Au, gold; SiN_*x*_, silicon nitride; EDC, 1-ethyl-3-(3-dimethylaminopropyl)carbodiimide; sulfo-NHS, *N*-hydroxysulfosuccinimide; PSA, prostate-specific antigen; PLL-PEG, poly-l-lysine-*grafted*-poly(ethylene glycol).

The principle of the new fabrication approach to give three-dimensional gold metallized nanopores embedded in free-standing silicon nitride (SiN_*x*_) membranes is outlined in Scheme 1A. Si(100) wafer, 105 μm-thick, sandwiched by low pressure chemical vapor deposition SiN_*x*_ layer on both sides, were employed as the starting material. Chips with free-standing membranes in the center were produced by semiconductor microfabrication techniques. The thinned SiN_*x*_ membrane (∼90 nm thick) was spin-coated with hexamethyldisilazane and electron beam resist ZEP520A in sequence. The deposited resist film, functioned as the electron beam drawing material during the electron beam lithography (EBL) process, as well as the masking material during the thermal metal deposition in a later process. Next, a dot array pattern was acquired by EBL at the ZEP520A resist film on the SiN_*x*_ membrane. Thereafter, the dot pattern was transferred into the underlying SiN_*x*_ membrane by reactive ion etching. As all regions of the chip, apart from the opened nanopores in the SiN_*x*_ membrane, were covered by the resist film, a titanium adhesion layer and gold film were deposited directly onto the nanopore surface by thermal evaporation under high vacuum condition. Finally, the ZEP520A resist film was lifted off in *N*-methyl pyrrolidone at 80 °C. By doing so, nanopore chips containing gold metallized nanopores in SiN_*x*_ membranes can be manufactured. More importantly, a material contrast between the nanopores and surrounding membranes is established. Therefore, it provides the opportunity to site-selectively modify the metallic nanopore using alkanethiol chemistry to bind to the gold whilst the SiN_*x*_ areas could subsequently be modified with an oxide compatible surface chemistry.^16^ The application of functionalized nanopore chips was demonstrated through its development as an ultrasensitive biosensor for diagnosing the cancer-related protein biomarker, PSA. Scheme 1B presents the surface modification procedure to build up functionalized nanopore chips through immobilizing anti-PSA antibodies onto nanopore surfaces and poly-l-lysine-*grafted*-poly(ethylene glycol) (PLL-PEG) on the rest of the membrane surface.

**Fig. 1:**
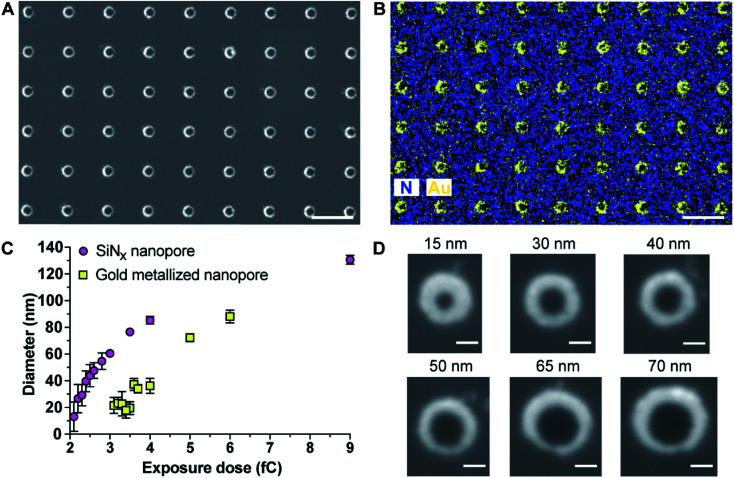
(A and B) Scanning electron microscope (SEM) image and elemental mapping of gold metallized nanopores by a FEI Nova SEM 450 equipped with a Bruker Silicon Drift energy dispersive spectroscopy (EDS) system. The EDS mapping of nitrogen and gold over the surface clearly indicates that gold coating was spatially deposited onto nanopores. Scale bar, 500 nm. (C) Dependence of the fabricated nanopore diameter, for SiN_*x*_ nanopores and gold metallized nanopores, upon electron beam exposure dose. Error bars represent standard deviations. (D) Representative enlarged-magnification of SEM images of gold metallized nanopores in different sizes. Scale bar, 50 nm.

The as-prepared gold metallized nanopores in SiN_*x*_ membranes were characterized by SEM in backscattered electron imaging mode. The SEM image shows an array of nanoscopic pores with the presence of “bright rings” (Fig. 1A). The energy dispersive spectroscopy (EDS) mapping over the surface confirmed that the bright rings were the deposited layer of gold coating on nanopores (Fig. 1B). Together these results show that the experimental realization of producing gold metallized nanopores embedded in a free-standing SiN_*x*_ membrane using the reported fabrication strategy was achieved. In addition, the metallic nanopores displayed a sharp contrast from the SiN_*x*_ membrane, which confirms the masking by the ZEP520A resist film during metal deposition process. Importantly, the thickness of gold coating can be tailored with nanoscale resolution using a quartz crystal microbalance-based monitor. To demonstrate the capability of fabricating metallic nanopores in varied diameters, a range of opened pores in different sizes on the resist film coated SiN_*x*_ membrane were produced by adjusting electron beam exposure doses during EBL. Thereafter, a layer of gold film (20–30 nm) was deposited onto the opened pores. The fabricated metallic nanopores were imaged by SEM and then the pore dimensions in the obtained SEM images were analyzed by ImageJ software. Fig. 1C shows the dynamic range of nanopore diameters of SiN_*x*_ and gold metallized nanopores on the membrane after exposing to a series of electron beam doses. The results showed that gold metallized nanopores can be produced in a range from few tens of nm to near 100 nm under applied conditions. Representative enlarged-magnification SEM images of metallic nanopores were presented in Fig. 1D. These images indicate that this fabrication approach is facile and robust in producing nanopores with tuneable diameters. In addition, Fig. S1 in the ESI† provides three representative high-resolution TEM images of metallic nanopores in different sizes.

**Fig. 2:**
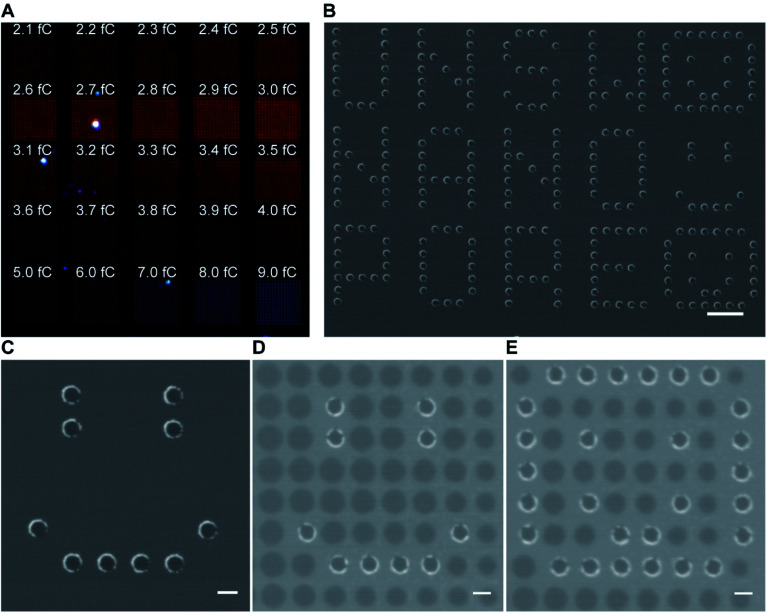
(A) Dark-field optical microscope image of a SiN_*x*_ membrane containing 25 arrays of 15 × 15 gold metallized nanopores of a variety of nanopore diameters. The nanopore diameter in each array increases gradually from top left to bottom right. (B and C) SEM images of gold metallized nanopores in a SiN_*x*_ membrane. Scale bars, 1 μm in (B); 200 nm in (C). (D and E) SEM images of a SiN_*x*_ membrane with the presence of both SiN_*x*_ nanopores and gold metallized nanopores. Scale bars, 200 nm.

As gold is well known to present localized surface plasmon resonance,^17^ 25 arrays of 15 × 15 gold metallized nanopores in SiN_*x*_ membrane were fabricated and then imaged by dark-field optical microscopy (Fig. 2A). The exposed electron doses for individual nanopore arrays were indicated in the image as well. The dark-field optical microscope image revealed a red shifting of scattering light from bigger nanopores (lower right) to smaller ones (top left), due to the localized surface plasmon resonant effect. Note that an electron beam exposure dose of 2.3 fC during EBL usually relates to the production of openings with a mean diameter of 30 nm on the SiN_*x*_ membrane. This means that openings in SiN_*x*_ membrane fabricated with a smaller electron exposure dose (≤2.3 fC) would result in the nanopore being fully blocked by the deposited gold. Hence, most of the imaged dots in the first arrow from the image were fully closed metallic nanopores. We speculate that nanopore devices with gold metallized nanopores may find the potential to couple nanoplasmonics into this platform as these metallic nanopores are both optically and electrically active.^18^ In addition to this, the fabrication approach described here was further demonstrated to be able to not only position gold metallized nanopores in SiN_*x*_ membranes with a high spatial resolution (matrix displaying LETTER and “SMILING” shapes as shown in Fig. 2B and C), but also produce nanopore arrays including metallic and semiconducting nanopores on a single membrane (Fig. 2D and E). Multiple operation on the SiN_*x*_ membrane is applicable to write and produce gold metallized nanopores as well as SiN_*x*_ nanopores with a full control over electron exposure dose and spatial location for individual nanopores. Taken together, these results clearly demonstrated that the fabrication approach presents good reproducibility and high-fidelity control over nanopore diameter, location, and combination.

We next presented the development of nanopore chips with gold metallized nanopores for ultrasensitive detection of PSA molecules. As mentioned, our previous study realized nanopore blockade sensing experiments using functionalized nanopore chips with 3 × 3 SiN_*x*_ nanopores and anti-PSA modified magnetic nanoparticles (abbreviated as (anti-PSA)-MNPs).^9^ Hereafter nanopore chips containing 3 × 3 gold metallized nanopores were fabricated and then chemically functionalized with the strategy depicted in Scheme 1B. Subsequently, nanopore blockade sensing of PSA molecules was performed with functionalized gold metallized nanopore chips and (anti-PSA)-MNPs under the same experimental conditions as used previously.^9^ The key difference between functionalized SiN_*x*_ nanopore chips and functionalized metallic nanopore chips was that all SiN_*x*_ nanopore chips were modified with one chemical functionality over the whole chip surface whilst the metallized nanopore chips presented two distinguished functionalities on nanopores and surrounding membranes, respectively. The core question is does this difference provide a distinct improvement in the nanopore blockade sensor at ultralow concentrations of analyte.

**Fig. 3:**
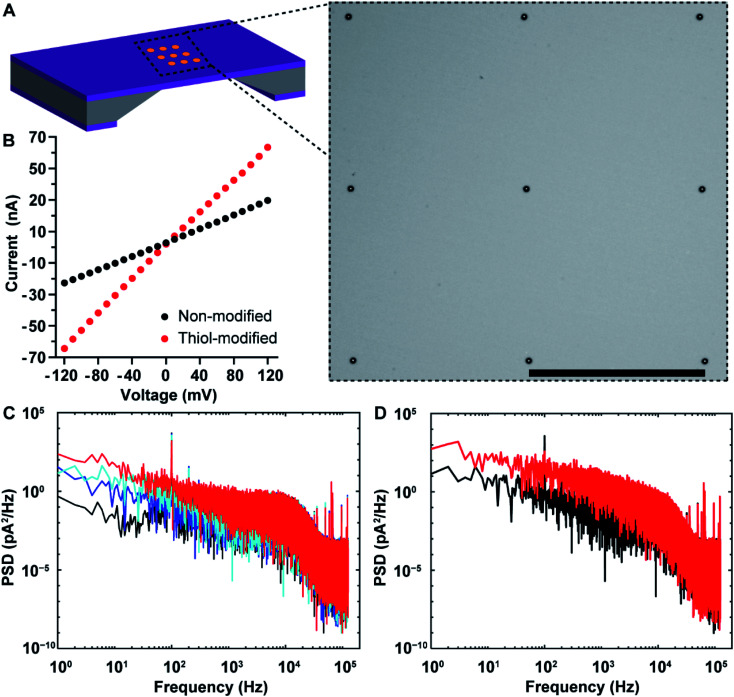
(A) Schematic illustration of a nanopore chip as well as a representative bright-field scanning transmission electron microscopy (STEM) image of SiN_*x*_ membrane containing an array of 3 × 3 metallic nanopores. Scale bar, 5 μm. (B) Current–voltage characteristic of a nanopore chip with 3 × 3 metallic nanopores before (black) and after (red) selective attachment of 1-mercapto-3,6,9,12-tetraoxapentadecan-15-oic acid onto gold nanopore surfaces. Electrolyte solution, 100 mM KCl (10 mM Tris–HCl, 0.05% Tween-20, pH 7.4). (C) The power spectral densities (PSDs) for a nanopore chip with non-modified 3 × 3 metallic nanopores under an applied potential differential of 0 mV (black curve), 50 mV (blue curve), 100 mV (cyan curve), and 150 mV (red curve), respectively. The ionic currents were acquired under a low-pass filter at 10 kHz, and a sampling frequency of 250 kHz. (D) The PSDs for a nanopore chip with 3 × 3 metallic nanopores before (black curve) and after (red curve) selective attachment of 1-mercapto-3,6,9,12-tetraoxapentadecan-15-oic acid onto the gold metallized nanopore surfaces. The ionic currents were acquired under an applied potential differential of 100 mV, a low-pass filter at 10 kHz, and a sampling frequency of 250 kHz.

Nanopore chips with a designed 3 × 3 array of 30 nm-diameter gold metallized nanopores spaced by a 5 μm distance were fabricated. A representative electron micrograph on the nanopore-containing membrane is provided in Fig. 3A. The resistance, and thus nanopore diameter, were estimated electrically from current–voltage (*I*–*V*) characteristic curves. Fig. 3B shows an example of the *I*–*V* curve obtained from a non-modified 3 × 3 metallic nanopore chip. The value of resistance was calculated from the current–voltage curve. With the obtained resistance and the Hille and Hall's model,^19–21^ the mean sizes of the bottom (*trans*) and topside (*cis*) orifices were estimated to be 33.5 nm and 99.0 nm, respectively. Here, assumptions of a 90 nm-thick SiN_*x*_ substrate membrane and a 70° sidewall angle were adopted. This dimensional estimation confirmed the successful fabrication of nanopore chips as designed, as well as the similarity of nanopore diameters for metallic nanopores to the SiN_*x*_ nanopores utilized in the previous work.^9^

After completing the characterization of nanopore diameters, we performed surface functionalization on the as-prepared 3 × 3 gold metallized nanopore chips. To probe changes in metallic nanopore surface, current–time recordings were obtained and power spectral density (PSD) of ionic current noise was calculated with a periodogram algorithm on 1 s current–time traces. Fig. S2A in the ESI† provides the schematic of main sources in PSD noises including four components, *i.e.*, flicker (also referred to as 1/*f*), thermal, dielectric and capacitance noises.^22,23^ First, a potential bias was applied across the nanopore chip, which led to an increased 1/*f* noise and a steeper slope of the 1/*f* dependent noise along with increasing the potential (Fig. 3C). This also confirmed that the metallic nanopores were opened, connecting the electrolyte solution in the *cis*-side to the *trans*-side chamber of the flow cell that were separated by the nanopore chip. Ionic flux, and the variability in ionic flux through the nanopore, have been suggested to be the main contributors to the low-frequency 1/*f* noise.^24,25^ Furthermore, PSD noises for ionic currents low-pass filtered at 1 kHz and 10 kHz are provided in Fig. S2B in the ESI.† Next, 1-mercapto-3,6,9,12-tetraoxapentadecan-15-oic acid was used to specifically modify the gold metallized nanopore surfaces. After coating with alkanethiols, the conductance of the modified 3 × 3 nanopores increased (Fig. 3B). An increased noise at frequencies (<2 kHz) was observed, which aligned well with the expectation that a self-assembled alkanethiol film on metallic nanopore surface would elevate the noise level, compared to that on the same chip of non-modified metallic nanopores (Fig. 3D). Dynamic fluctuations of the immobilized molecules in the nanopores have been suggested to be responsible for the increased 1/*f* noise.^25–27^ Finally, the distal carboxyl groups on the alkanethiol modified nanopore surface were activated and then conjugated with amine groups on anti-PSA antibodies. The surface chemical composition on gold metallized nanopores was denoted as thiol-PEG_4_-(anti-PSA). That is, anti-PSA antibodies were spatially immobilized onto the nanopore surface. Meanwhile, PLL-PEG coating was electrostatically deposited onto surrounding membranes, suppressing nonspecific adsorption on the membrane. This selective immobilization of molecules onto gold and SiN_*x*_ has been demonstrated previously on planar surfaces.^16^ The chemical functionalities on the functionalized nanopore chips can be removed *via* oxygen plasma treatment and regenerated for multiple times. There are only a few attempts^28–31^ at obtaining site-selective modification on solid-state nanopores that typically use colloidal lithography to fabricate the nanopores and either used sacrificial layers to place surface chemistry on one location and not the other^28^ or had a layered structure of two materials such that there were well defined layers of surface chemistry within the nanopore.^29,30^ The fabrication approach reported herein is a further advance over these capabilities because firstly it can produce ordered arrays of nanopores where the fabrication approach presents the control over nanopore numbers, and diameters and positions that are modulable for individual metallic nanopores or even with a mixture of metallic and semiconducting nanopores, the entire inner pore walls can be modified with the same surface chemistry which is different to the exterior surface and as there is no further fabrication processing even biological and delicate chemistries can be used. Importantly, there is a clear material contrast between nanopore interior and exterior in gold metallized nanopore chips. This ensures that a straightforward surface modification approach can be utilized by using alkanethiol and silane chemistries on the gold metallized nanopores and silicon nitride membranes, respectively. This functionalization approach is compatible with chips containing nanopore arrays. Additionally, the metallic nature of the gold metallized nanopore is expected to be chemically stable against slow etching by electrolytes.

It is noted that previous studies have reported the fabrication of gold nanotubules in polymeric membranes (*e.g.*, polycarbonate) of a thickness in micron scale.^32–36^ The polycarbonate filters with damage track undergo a chemical etching process to create pores that span the entire thickness of the membrane. Afterwards, gold nanotubules are prepared *via* electroless deposition of gold onto the surfaces of and the pore walls of the polycarbonate filtration membrane. Typically, a strip of Scotch tape or ethanol-wetted cotton swab is used to remove the surface gold films at the membrane, leaving gold nanotubules in the membrane of microsized thickness. In contrast, the work reported here represents a new development that allows not only an unprecedented control over locations and dimensions of individual nanopores, but the fabrication approach is compatible with thermal metal deposition thus multiple samples can be prepared on one go. Moreover, a standard lift-off process is utilized to obtain chips containing gold lined nanopores. Importantly, these gold lined nanopores span thin substrate membranes (about 90 nm thick) and the ratio of the pore openings on the two sides of the membrane is small (less than 5), compared with that in thick gold nanotubule membranes having a ratio of pore openings more than 20. Additionally, under the unique nanopore blockade sensing paradigm, the capability of electrically sensing single molecule detection events in parallel at chips with an array of multiple gold lined nanopores is realized.

**Scheme 2:**
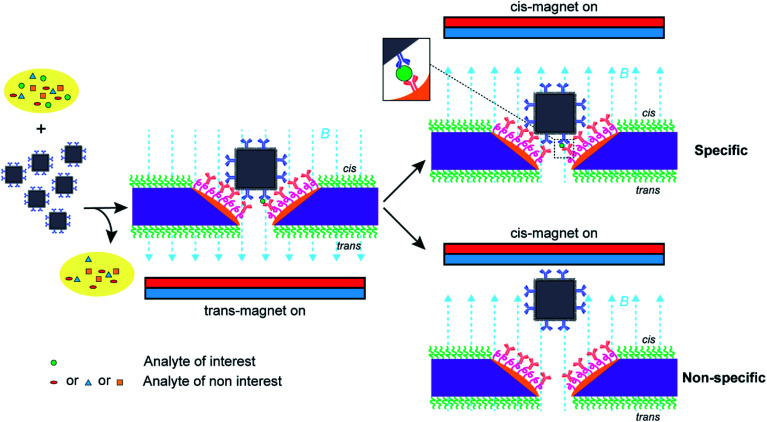
Nanopore blockade sensing using functionalized nanopore chips with spatially distinguished functionalities and anti-PSA modified magnetic nanoparticles ((anti-PSA)-MNPs). The (anti-PSA)-MNPs are employed to capture target PSA molecules from the sample, washed three times to remove nonspecific molecules, and then introduced into the electrolyte solution in the *cis*-side chamber of a flow cell and driven to the nanopores under a *trans*-magnet followed by the application of a *cis*-magnet to differentiate specific and nonspecific blocking events for the specific detection of PSA molecules for analytical analysis.

**Fig. 4:**
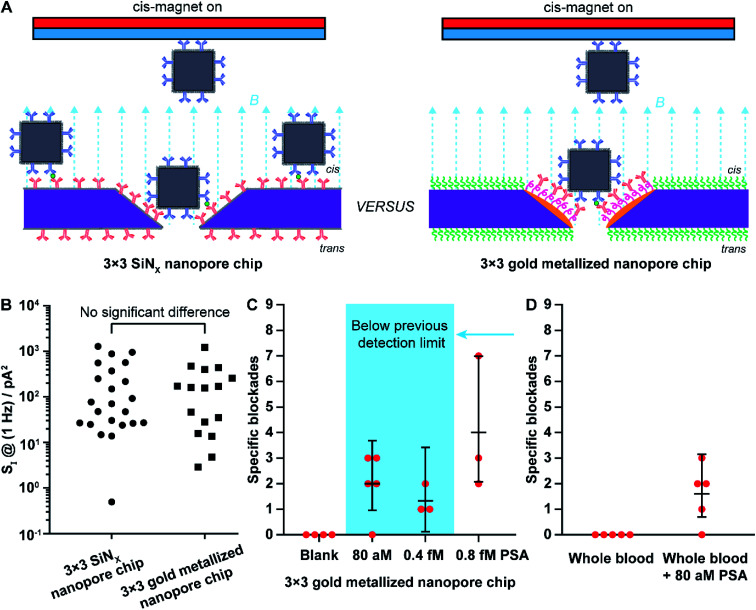
(A) Scheme of nanopore blockade sensing with functionalized chips of 3 × 3 SiN_*x*_ nanopores and functionalized chips of 3 × 3 gold metallized nanopores, respectively. (B) Comparison of the low-frequency 1/*f* noise between functionalized 3 × 3 SiN_*x*_ nanopore chips and functionalized 3 × 3 gold metallized nanopore chips. Each data point is extracted from an independent nanopore chip. Kruskal–Wallis *H* test using SPSS Statistics software (Version 26) indicates that there is no significant difference in the shape of the distribution of 1/*f* noise at 1 Hz (S_I_ @ 1 Hz) between 3 × 3 SiN_*x*_ nanopore chips and 3 × 3 gold metallized nanopore chips. (C) Mean values of detected specific nanopore blockades for PSA molecules in different concentrations using functionalized 3 × 3 metallic nanopore chips. (D) Comparison of mean value of specific nanopore blockades determined with functionalized 3 × 3 gold metallized nanopore chips in whole blood with and without 80 aM PSA spiked. Briefly, 4 μL solution of (anti-PSA)-MNPs was mixed with whole blood (16 μL) for about 45 min to extract PSA from whole blood. Afterwards, the (anti-PSA)-MNPs were magnetically separated and washed three times to remove non-specifically adsorbed molecules followed by adding to 100 mM KCl solution (10 mM Tris–HCl, 0.05% Tween-20, pH 7.4) to perform magnetic analyte shuttling for sensing 80 aM PSA. The 95% confidence intervals for Poisson means are calculated by the exact method. Each data point was obtained from an independent nanopore chip.

Nanopore blockade sensing utilizes a cycle of 10 min *trans*-magnet to bring (anti-PSA)-MNPs to the nanopores and then 5 min *cis*-magnet to pull (anti-PSA)-MNPs out of the sensing surface (see Scheme 2). The antibody on the (anti-PSA)-MNPs is raised against the epitope 5 of PSA. If the (anti-PSA)-MNPs have captured PSA molecules, then they can form an immunosandwich with the anti-PSA antibodies immobilized in the nanopore raised to the epitope 1 of PSA, and as such the reversal of the magnetic field does not result in the unblocking of the nanopore. In contrast, if no PSA is captured the (anti-PSA)-MNPs will be removed on application of the *cis*-magnet. A total of 4 such cycles of magnet switching is typically involved to perform a biosensing experiment. As illustrated in Fig. 4A, when working with 3 × 3 SiN_*x*_ nanopore chips functionalized with a single surface chemistry denoted as silane-PEG_6_-(anti-PSA),^9^ (anti-PSA)-MNPs with captured PSA molecules can bind to both the nanoscale pore surface and the much wider surrounding membrane under the *trans*-magnet. According to our previous computational modeling,^9^ apart from the (anti-PSA)-MNPs entering the nanopores directly from solution to cause the nanopore blockades, (anti-PSA)-MNPs can also take an indirect entry approach to enter the nanopores. That is (anti-PSA)-MNPs, after being magnetically attracted to the *cis* membrane, if they land within the vicinity of a nanopore (within 150 nm), they can then be attracted toward the nanopore electrophoretically.^9^ Hence, it is anticipated that a greater proportion of (anti-PSA)-MNPs with captured PSA molecules would be lost due to the binding to anti-PSA antibodies on the *cis* membrane such that they can never reach the nanopore to give a signal. In contrast, with a layer of PLL-PEG coating on the *cis* membrane to limit nonspecific adsorption, more nanoparticles would be expected to come into nanopores at nanopore chips modified with spatially differentiated functionalities. This would help to reduce the loss of nanoparticles on the *cis* membrane, confine specific recognition events occurred in nanopores, and lead to a higher number of specific nanopore blockades to be detected which is helpful to lower detection limits.

First, the 1/*f* dependent noises at 1 Hz (S_I_ @ 1 Hz) were obtained as an indicator of noise levels^37^ for 3 × 3 SiN_*x*_ nanopore chips and 3 × 3 gold metallized nanopore chips. Statistical analysis indicated that the distribution of the 1/*f* noise at 1 Hz for the two types of nanopore chips was not significantly different, regardless of the difference in nanopore material (Fig. 4B). Next, nanopore blockade sensing of PSA was performed. We started with the measurement of 0.8 fM PSA which is the lowest detectable PSA concentration with 3 × 3 SiN_*x*_ nanopore blockade sensors.^9^ The fractions of (anti-PSA)-MNPs with 0, 1, and 2 (or more) bound PSA molecules in all 1.87 × 10^9^ (anti-PSA)-MNPs are 99.98%, 0.019% and 1.9 × 10^−6^%, respectively. Statistically each specific nanopore blockade is attributed to single-molecule PSA detection event. As shown in Fig. 4C, for the four magnetic switching cycles, the mean value of detected specific nanopore blockades for 0.8 fM PSA was 4.0 counts at gold metallized nanopore chips with the localized thiol-PEG_4_-(anti-PSA) capture chemistry on nanopores. This compares favorably to the SiN_*x*_ nanopore chips with the silane-PEG_6_-(anti-PSA) capture chemistry over the whole chip surface where 2.7 counts were achieved.^9^ These results indicate that more nanoparticles reached the nanopores along with more specific blockades detected, showing the advantage of using nanopore chips with site-specific functionalities. Representative SEM images on these two kinds of functionalized nanopore chips after PSA sensing experiments are presented in Fig. S3 in the ESI.†

Considering the higher number of capture events for the gold metallized nanopore chips with capture antibodies only in the nanopore, over the SiN_*x*_ nanopore chips with antibodies everywhere, we next investigated whether an even lower PSA detection limit than the previously reported 0.8 fM was possible. The 3 × 3 gold nanopore blockade sensors showed an averaged 1.3 specific blockades for 0.4 fM PSA and 2.0 specific blockades for 80 aM PSA. A representative ionic current trace for sensing 80 aM PSA and examples of blocking and unblocking events are presented in Fig. S4 and S5.† Clear steps in current–time traces indicating the blocking and unblocking of nanopores can be observed and counted although the step amplitude varies to some extent. Note that with only 9 nanopores embedded in the chip, the nanopore blockade sensor may not present a sensitivity enough to discriminate these two concentrations. It clearly demonstrated that with the implementation of the refined surface functionality strategy on gold nanopore chips, nanopore blockade sensors are capable of selectively detecting PSA molecules in ultralow concentrations. Thus, the simple change in surface chemistry so that capture antibodies were only in the nanopore decreased the lowest detected concentration by an order of magnitude. It is important to note at these low concentrations of analyte, the nanopore blockade events are a low probability event with only 9 nanopores, such that uncertainties are large, and hence the nanopore blockade sensor near the lowest detected concentration is only semi-quantitative. Fig. 4C however does show that with site selective surface chemistry the sensor responds to lower concentrations, which is of great significance for early diagnosis of diseases and monitoring of disease reoccurrence.^38^ The robustness of the 3 × 3 gold nanopore blockade sensor was further demonstrated by the sensing of 80 aM PSA extracted from whole blood (Fig. 4D). Similar results were obtained, compared to the measurements of PSA extracted from buffer solutions. It was also confirmed that no specific blockades were observed in the absence of PSA extracted from buffer solutions and whole blood. Taken together, these results indicate the advantageous employment of a differentiated surface functionality strategy to nanopore chips in the development of nanopore based ultrasensitive biosensors.

## Conclusions

In conclusion, this study has successfully established the fabrication approach to produce gold metallized nanopores in SiN_*x*_ membranes, which presents the capacity to modulate nanopore number, diameter, and combination. Importantly, this is the first report on the fabrication of entirely three-dimensional metallic nanopore structures incorporated into a solid-state thin membrane. The choice of deposited metal is not limited to gold. The fabrication approach described here may provide a promising strategy to fabricate nanopores metallized by other metals that are compatible with evaporation process. Furthermore, it was shown the observation of localized surface plasmon resonance effect from gold metallized nanopores in the SiN_*x*_ membrane as well as a red shifting of the scattered light on the metallic nanopore along with decreasing the nanopore diameter. In addition, it was shown here that localized surface functionalization on nanopore surfaces and surrounding membranes can be realized. This selective surface modification is attributed to the adopted orthogonal self-assembly methodology using alkanethiol chemistry and an oxide compatible surface chemistry to modify gold and SiN_*x*_ surfaces, respectively. Moreover, the advantage of using nanopore chips modified with spatially differentiated functionalities in nanopore sensing was demonstrated by the ultrasensitive detection of a model cancer-related protein biomarker, PSA. Nanopore blockade sensing at functionalized 3 × 3 gold metallized nanopore chips including refined chemical functionalities was shown to be able to lower the detectable concentration of PSA molecules by one order of magnitude to as low as 80 aM, compared to that from 3 × 3 SiN_*x*_ nanopore chips modified with a single chemical functionality. This study reports the capability to fabricate gold metallized nanopores that can be optically and electrically active, as well as site-specific surface modification that enables a refined control of surface chemistries on nanopore devices. Thus, we envision that nanopore sensors built up upon solid-state gold metallized nanopore chips may find further developments including optical nanopore sensing, multiplexed detection, and direct processing with complex biological samples.

## Author contributions

Y. W. and J. J. G. conceived and supervised the project; Y. W. designed and performed the experiments; Y. Y. contributed to the SEM imaging in backscattered electron imaging mode; S. C. contributed to the TEM imaging; Y. W. wrote the original draft and J. J. G. helped with revising the manuscript; and all authors commented on the manuscript. All authors approved the final version.

## Conflicts of interest

The authors declare no conflict of interest.

## Supplementary Material

SC-011-D0SC04552G-s001
