# Supplementary material for: Selectively detecting attomolar concentrations of proteins using gold lined nanopores in a nanopore blockade sensor
Source: Chem Sci. 2020 Oct 26;11(46):12570–9. doi: 10.1039/d0sc04552g (PMC8163308; doi:10.1039/d0sc04552g)
Supplement: SC-011-D0SC04552G-s001 [file SC-011-D0SC04552G-s001.pdf]

Electronic Supplementary Material (ESI) for Chemical Science.
This journal is © The Royal Society of Chemistry 2020

Electronic Supplementary Information

Selectively detecting attomolar concentrations of proteins using gold lined nanopores in a nanopore blockade sensor

Yanfang Wu[1,*], Yin Yao[2], Soshan Cheong[2], Richard D. Tilley[1,2], J. Justin Gooding[1,*]

[1] School of Chemistry, Australian Centre for NanoMedicine, and Australian Research Council Centre of Excellence in Convergent Bio-Nano Science and Technology, University of New South Wales, Sydney, New South Wales 2052, Australia

[2] Electron Microscope Unit, Mark Wainwright Analytical Centre, University of New South Wales, Sydney, New South Wales 2052, Australia

* Correspondence: yanfang.wu@unsw.edu.au (Y. Wu), justin.gooding@unsw.edu.au (J. J. Gooding)

Table of Contents

1. Experimental Section

1.1 Chemicals and Materials

1.2 Fabrication of Nanopore Chips Containing Gold Metallized Nanopores

1.3 Site-Selective Functionalization on Nanopore Chips Containing Gold Metallized Nanopores

1.4 Ionic Current Measurements and Analysis

1.5 Apparatus

2. Supplementary Figure S1–S6

3. References

1. Experimental Section

1.1 Chemicals and Materials

Unless otherwise stated, all chemicals were at least of analytical reagents grade and used as received. Milli-Q water (~18 MΩ cm, Millipore, Australia) was used for preparing aqueous solutions in this study. Prime grade double-side polished undoped silicon (100) wafers (105 μm thick, >20 Ω cm resistivity) coated with 180-nm-thick low pressure chemical vapour deposition (LPCVD) silicon nitride (SiN_x) film on both sides were purchased from Virginia Semiconductor Inc. (Virginia, USA). Human prostate specific antigen (PSA), mouse monoclonal anti-PSA antibody specific to epitope 1 of PSA (catalog number ab10187) and mouse monoclonal anti-PSA antibody specific to epitope 5 of PSA (catalog number ab10185) were provided by Abcam and purchased from Sapphire Bioscience (Sydney, Australia). The dissociation constant (K_D) for anti-PSA antibody (catalog number ab10187) and PSA was reported to be 2.17×10^{-8} M while the K_D for anti-PSA antibody (catalog number ab10185) and PSA was 1.75×10^{-8} M.[1] Whole blood was ordered from Australian Red Cross.

1.2 Fabrication of Nanopore Chips Containing Gold Metallized Nanopores

SiN_x membranes were produced from Si(100) wafers sandwiched with 180-nm-thick LPCVD SiN_x films by conventional micro-fabrication techniques firstly. The backside of one wafer was coated with hexamethyldisilazane and AZ nLOF2020 photoresist at 3000 revolutions per min (rpm) for 30 s, respectively and prebaked at 110 °C for 1 min; square window patterns were created by photolithography in the photoresist film. After a 1 min post-exposure bake at 110 °C, the exposed resist film was developed in AZ826 MIF developer for 1 min, rinsed with Milli-Q water for 30 s and blown dry with nitrogen stream. The exposed SiN_x layer was removed in a reactive ion etching (RIE) SiN_x etching process (PlasmaLab system 100, Oxford Instrument Co., UK) using a mixture of argon and CF_4 gases (an etching time of 360 s). Wet chemical etching was performed in 45% potassium hydroxide solution at 85 °C to obtain free-standing SiN_x membrane. Afterwards, SiN_x membranes on the frontside of chips were thinned down to ~90 nm thick using RIE. Next, hexamethyldisilazane and ZEP520A resist (Nippon Zeon Co.,

Japan) was subsequently spun onto the thinned SiN_x membrane on a chip (5000 rpm for 30 s) and annealed for 2 min at hot plate of 180 °C. Dot patterns were produced with a Raith 150^{TWO} system (Raith GmbH, Germany). The dot patterns were developed in *N*-amyl acetate for 2 min, rinsed with methyl isobutyl ketone for 30 s and dried with a stream of nitrogen. A second RIE SiN_x etching process was performed to transfer the dot patterns into the free-standing SiN_x membrane. Then metal coating of the ZEP520A coated chips with drilled SiN_x nanopores was carried out using thermal vapour deposition of titanium and gold onto nanopore interiors. Deposition was performed in high vacuum ($<3\times10^{-6}$ Torr) at a thermal evaporator equipped with a bell jar (Kurt J. Lesker Company, USA). The deposition rate and film thickness were monitored by a quartz crystal microbalance. Finally, the ZEP520A resist film was lifted off in *N*-methyl pyrrolidone at 80 °C and the chips were cleaned by oxygen plasma treatment for 5 min.

1.3 Site-Selective Functionalization on Nanopore Chips Containing Gold Metallized Nanopores

Material specific modification strategy was employed to functionalize nanopore chips with metallic nanopores in two steps.[2] Firstly, nanopore chips of metallic nanopores were inserted into ethanolic solution of 0.5 mg mL^{-1} 1-mercapto-3,6,9,12-tetraoxapentadecan-15-oic acid overnight under room temperature and rinsed with anhydrous ethanol. Next, the modified chips with terminal carboxylic groups were activated by 1-ethyl-3-(3-dimethylaminopropyl)carbodimide (EDC) and *N*-hydroxysulfosuccinimide (sulfo-NHS) (200 mM/200 mM) in 0.1 M MES (2-(*N*-morpholino)ethanesulfonic acid) solution with a pH value of 6.0 for 30 min, followed with insertion in 0.1 M MES solution (pH 7.5) of anti-PSA antibodies (catalog number ab10187) for 60 min and rinsed with MES solution (0.1 M, pH 7.5). Finally, chips were immersed into phosphate buffered saline (1× PBS, pH 7.4, purchased from Sigma Aldrich, USA) solution of 0.05 mg mL^{-1} poly-*L*-lysine-grafted-poly(ethylene glycol) (PLL-PEG) for 30 min under room temperature and rinsed with PBS solution. Chips were stored in refrigerator at 4 °C prior to use.

1.4 Ionic Current Measurements and Analysis

Functionalized nanopore chips were sandwiched with two polydimethylsiloxane (PDMS) layers and mounted between two compartments of a custom-made flow cell filled with degassed and filtered (0.2 μm, Millipore, Germany) electrolyte solution of 100 mM KCl and 10 mM tris(hydroxymethyl)aminomethane (Tris-base, pH 7.4). Two Ag/AgCl electrodes were inserted into the flow cell and connected to an Axopatch 200B patch clamp amplifier (Axon Instrument, Molecular Devices, USA) in voltage clamp mode and a biased potential was applied between these two electrodes. Detailed experimental procedure is referred to our previous studies.[3-4] In brief, 20 μL aliquots of samples were prepared by mixing 4 μL solution of 1.87 × 10^9 (anti-PSA)-MNPs with 16 μL solution of PSA in different concentrations at room temperature for 45 min. Afterwards, the (anti-PSA)-MNPs were magnetically separated, washed three times and re-dispersed in 20 μL KCl electrolyte solution which was added into the electrolyte in the *cis*-side chamber of the flow cell. For measuring ionic currents at one nanopore chip, the *trans*-magnet was applied for 10 min followed with the *cis*-magnet for 5 min; this magnet switching repeats 4 times. Current-time traces were filtered with a built-in 4-pole low-pass Bessel filter (80 dB/decade) at 10 kHz and acquired with Clampex (Version 10.4, Axon Instrument, USA) via a DigiData 1440A (Axon Instruments, Molecular Devices, USA) analog-to-digital converter at 250 kHz sampling rate. Clampfit (Version 10.4, Axon Instruments, Molecular Devices, USA) was used to analyze current-time traces. The synthesis of cubic iron oxide magnetic nanoparticles (see Figure S6 for the characterization of nanoparticles) and subsequent attachment of anti-PSA antibody (catalog number ab10185) follow our previous studies.[3,5]

1.5 Apparatus

Electron beam lithography (EBL) was conducted to produce dot patterns using a Raith 150^{TWO} system with an acceleration voltage of 10 kV and a beam aperture of 10 μm. Nanopore chips containing gold metallized nanopores were characterized by a scanning electron microscopy (FEI Nova Nano SEM 450, USA) equipped with an energy dispersive spectroscopy (EDS) system and nanopore dimensions from the obtained SEM images were analyzed with ImageJ

software. Transmission electron microscopy (TEM) images were acquired on a JEOL JEM-F200 operated at 200 kV. Oxygen plasma treatment was carried out using PlasmaLab system 100 (Oxford Instrument Co., UK) with a radio frequency power supply of 100 W and a process temperature of 20 ^{o}C. Dark-field optical imaging was performed on an Olympus BX51 dark-field microscopy.

2. Supplementary Figures S1-S6

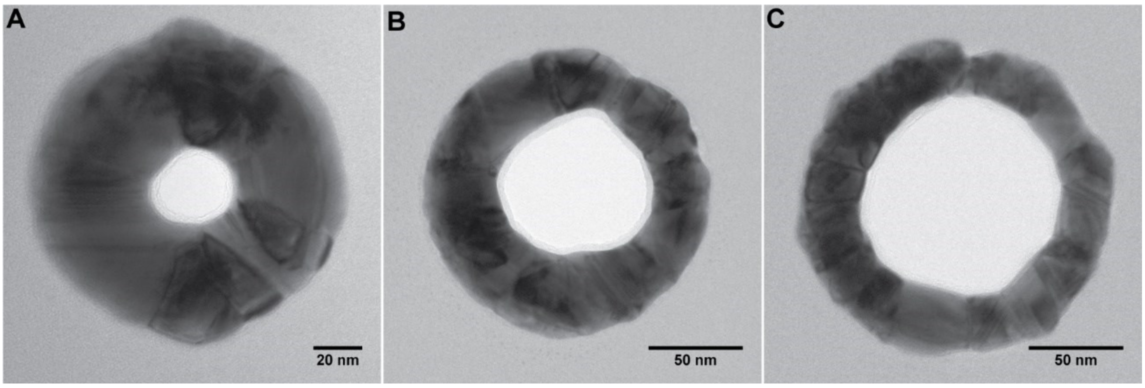

Figure S1. (A, B and C) Transmission electron microscopy (TEM) images of gold metallized nanopores in three different diameters.

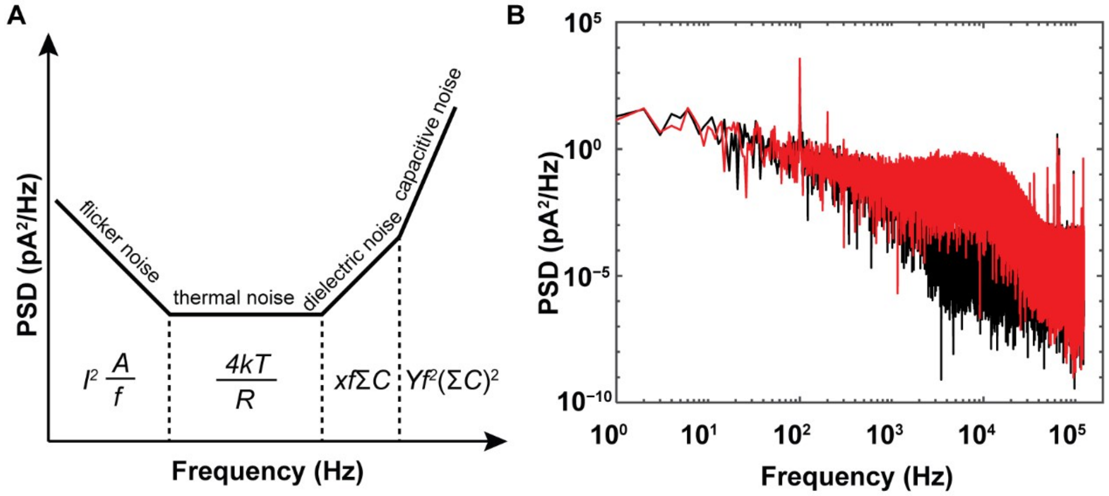

Figure S2. (A) Schematic of main sources of noises in power spectral density (PSD). (B) The noise PSDs for a nanopore chip containing 3×3 gold metallized nanopores low-pass filtered at a frequency of 1 kHz (black curve) and 10 kHz (red curve). The ionic currents were acquired with an Axopatch 200B patch clamp amplifier at 250 kHz sampling rate under an applied potential bias of 100 mV.

Current-time traces at a nanopore chip containing non-modified 3×3 metallic nanopores were recorded and the PSD noises were calculated using a periodogram algorithm from 1 s current-time traces. The PSD noises in ionic current experiments can be divided into two main regimes, the low-frequency regime, and the high-frequency regime. The flicker noise (1/f dependence) and thermal noise are dominant in the low-frequency PSD spectra while dielectric loss and capacitance noise are the two main contributors in the higher-frequency PSD spectra. In high-frequency regime (>40 kHz), a minimal capacitance noise was observed because of the employment of a built-in analog 4-pole low-pass Bessel filter. As expected, the noise increases along with the bandwidth (hertz, Hz) of the measurement (Figure S2).

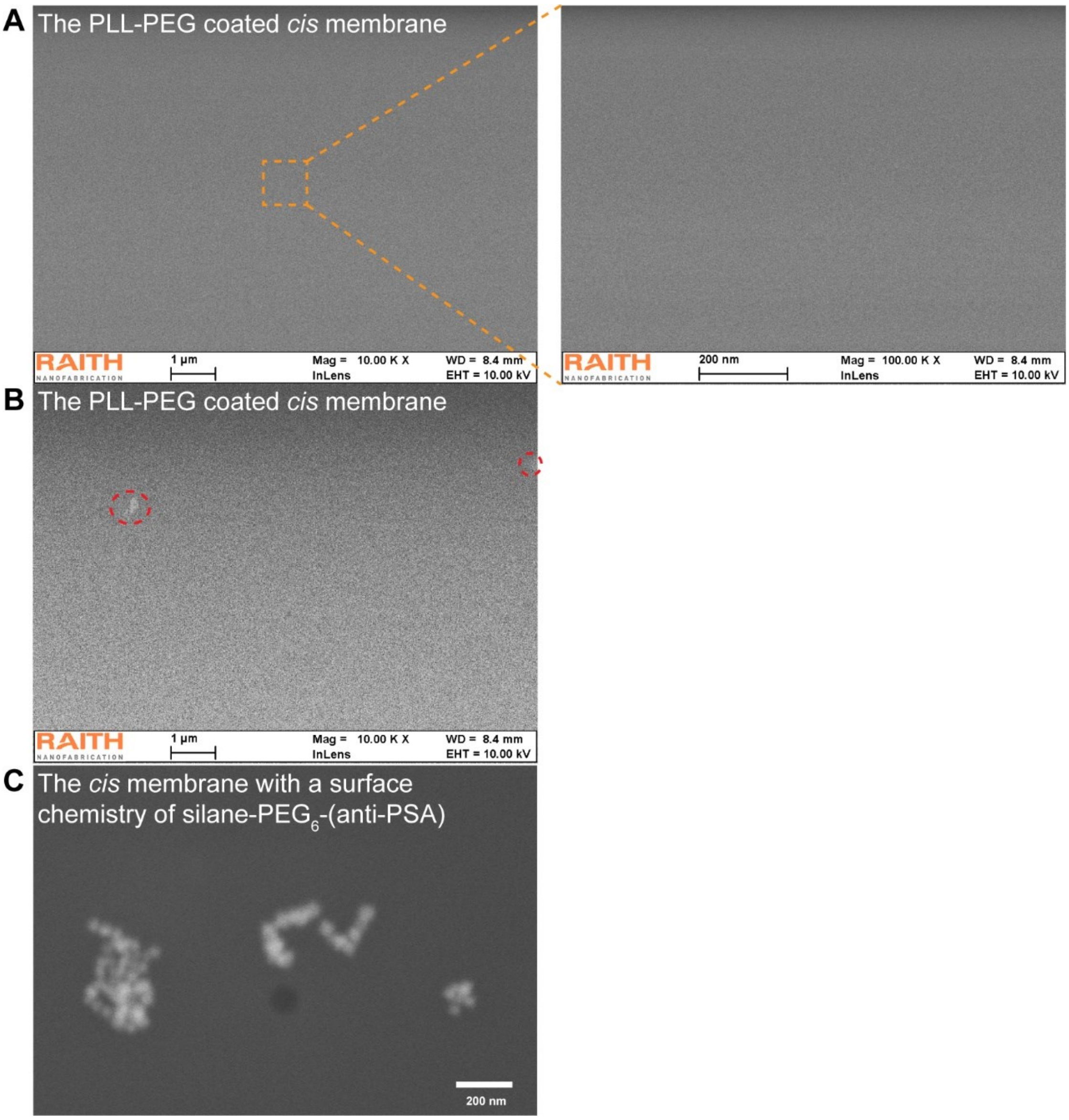

Figure S3. SEM images of (A, B) two functionalized nanopore chips containing 3×3 gold metallized nanopores and (C) one functionalized nanopore chip with 3×3 SiN_x nanopores after PSA sensing experiments. A zoom-in electron microscope image for the central region was provided to the right side of panel (A).

After PSA sensing experiments, three functionalized nanopore devices containing 3×3 gold metallized nanopores and one functionalized nanopore devices with 3×3 SiN_x nanopores were characterized by SEM. No (anti-PSA)-MNPs were found on the PLL-PEG coated *cis* membrane on two gold metallized nanopore chips. Representative images were presented in Figure S3(A). For the third gold metallized nanopore chip, very few (anti-PSA)-MNPs were observed on the PLL-PEG modified membrane from a large scale (Figure S3B). Together these results indicate a reasonably effective antifouling performance for the PLL-PEG coating against (anti-PSA)-MNPs. In contrast, on the functionalized SiN_x nanopore chip[2] presenting a surface chemistry of silane-PEG_6-(anti-PSA), some (anti-PSA)-MNPs were found outside of a SiN_x nanopore on the *cis* membrane (Figure S3C), suggesting that to some degree there tends to be a loss of (anti-PSA)-MNPs, indicating that.

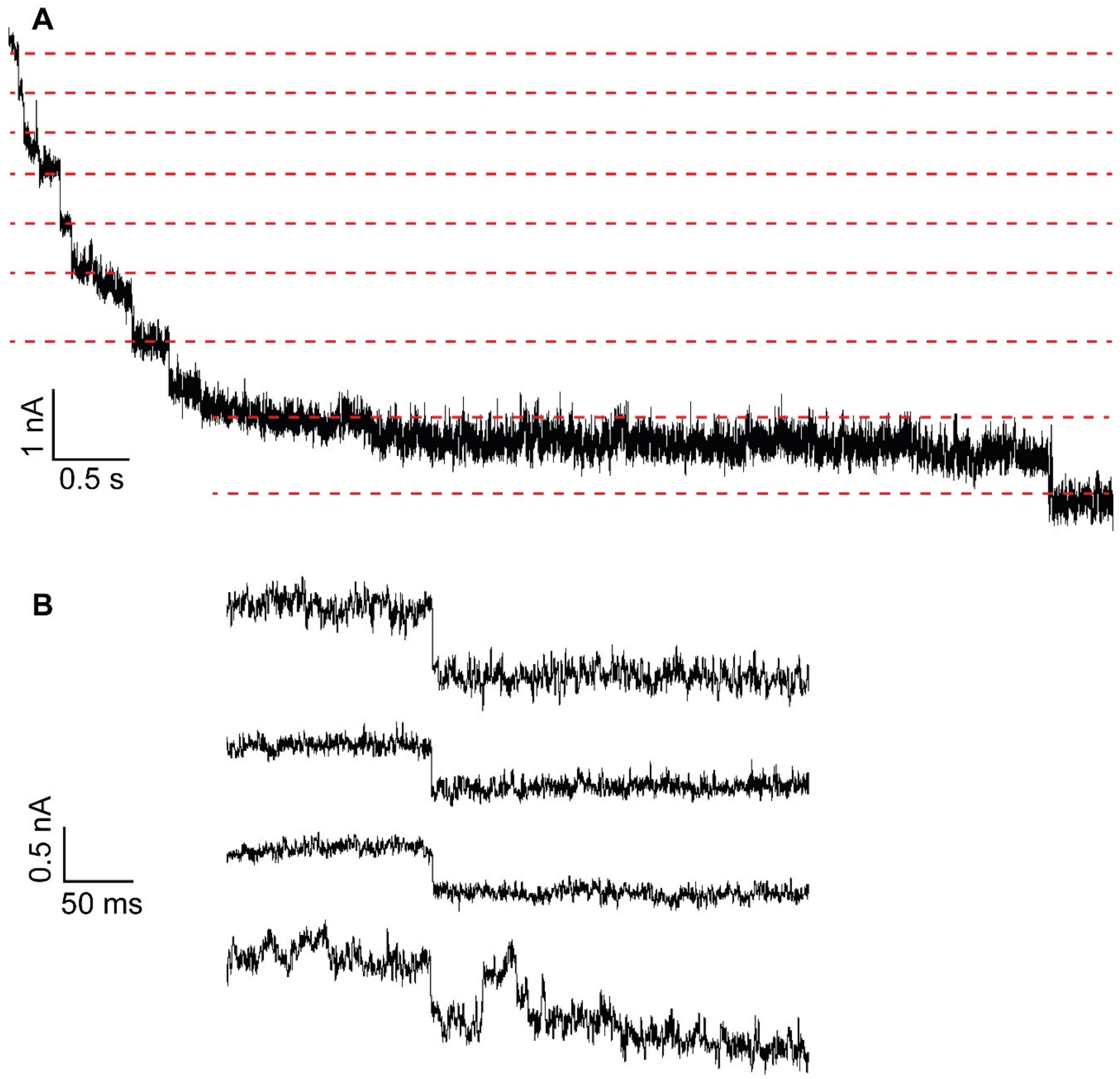

Figure S4. (A) A snapshot of the current-time trace from a functionalized nanopore chip with 3×3 gold metallized nanopores for the measurement of 80 aM PSA under a *trans*-magnet. A total number of 1.87×10^{9} (anti-PSA)-MNPs were employed to capture PSA molecules, magnetically collected, washed several times, and finally added to 100 mM KCl solution (10 mM Tris-HCl, 0.05% Tween-20, pH 7.4). The ionic current was acquired under an applied potential differential of 100 mV, a low-pass filter at 10 kHz, and a sampling frequency of 250 kHz. (B) Examples of current-time traces showing blockade events at functionalized nanopore chips containing 3×3 gold metallized nanopores.

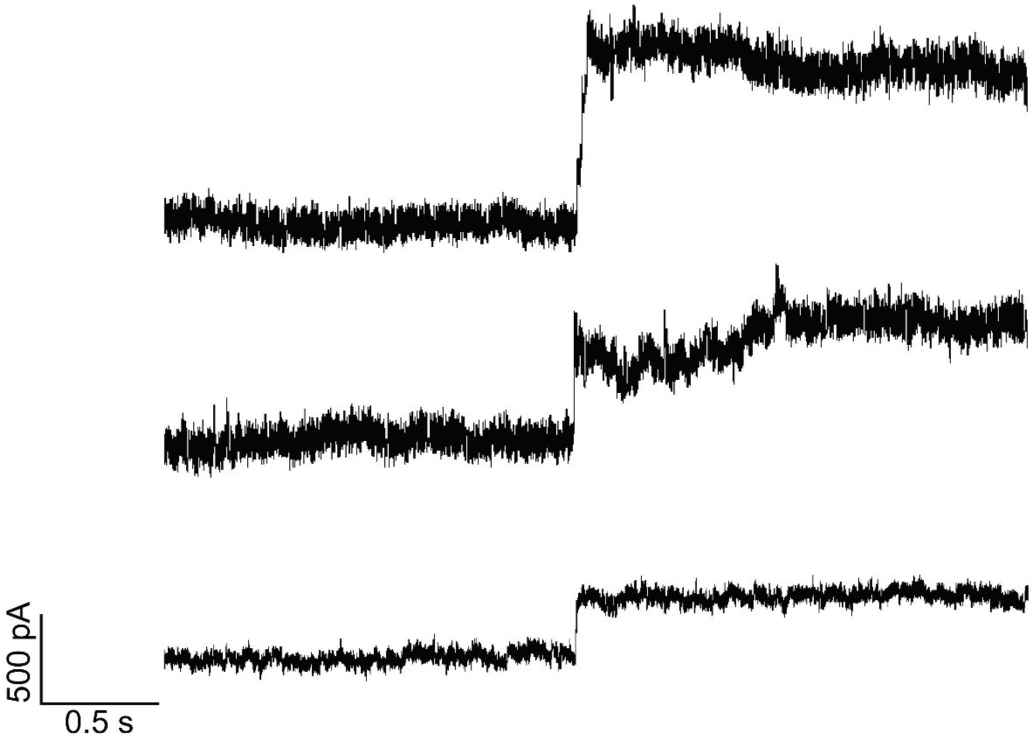

Figure S5. Representative current-time traces at functionalized nanopore chips with 3×3 gold metallized nanopores showing the unblocking of (anti-PSA)-MNPs from the nanopore under the *cis*-magnet.

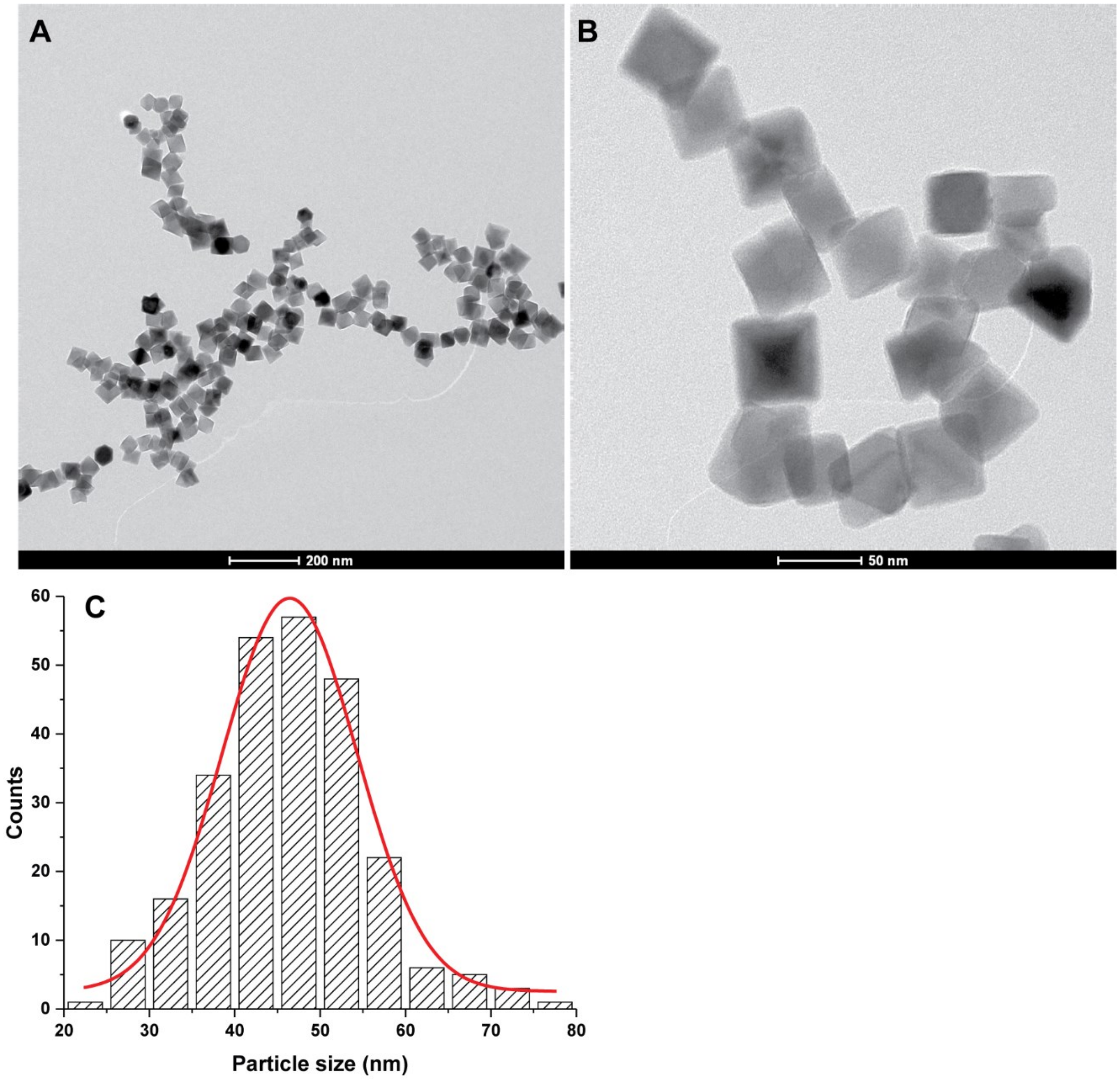

Figure S6. (A, B) Transmission electron microscopy images and (C) size distribution of polyethyleneimine modified magnetic nanoparticles. The average size of the nanoparticles was found to be (46.4±7.9) nm (Number of particles measured, N=257) and the nanoparticle polydispersity was 17%.

3. References

[1] P. Damborský, N. Madaboosi, V. Chu, J. P. Conde, and J. Katrlík, *Chemical Papers*, 2015, **69**, 143–149.

[2] Y. Wu, J. Lian, V.R. Gonçales, R. Pardehkhorram, W. Tang, R. D. Tilley, and J. J. Gooding, *Langmuir*, 2020, **36**, 5243–5250.

[3] K. Chuah, Y. Wu, S.R.C. Vivekchand, K. Gaus, P. J. Reece, A. P. Micolich, and J. J. Gooding, *Nat. Commun.,* 2019, **10**, 2109.

[4] Y. Wu, K. Chuah, and J. J. Gooding, *Biosens. Bioelectron.*, 2020, **165**, 112434.

[5] I. Y. Goon, C. Zhang, M. Lim, J. J. Gooding, and R. Amal, *Langmuir,* 2010, **26**, 12247–12252.
